# Preparation and Characterization of the Properties of Atmospheric Plasma-Sprayed Sr/Mg-Doped Bioactive Glass Coatings on Titanium Alloys

**DOI:** 10.3390/ma19173596

**Published:** 2026-08-24

**Authors:** Da Zeng, Yanwen Chen, Jianfeng Chen, Cijun Shuai, Fangwei Qi, Peilin Chen, Deping Wang

**Affiliations:** 1 School of Materials Science and Engineering, Tongji University, Shanghai 201804, China; 2 Double Medical Technology Inc., Xiamen 361026, China; 3 Jiangxi Province Key Laboratory of Additive Manufacturing of Implantable Medical Device, Jiangxi University of Science and Technology, Nanchang 330013, China; shuai@csu.edu.cn (C.S.); qfw@jxust.edu.cn (F.Q.)

**Keywords:** titanium alloys, bioactive glass, composition design, atmospheric plasma spray, osseointegration

## Abstract

Titanium alloys are widely used in clinical settings due to their excellent mechanical properties and biocompatibility. However, the biologically inert surface of titanium alloys limits interfacial bioactivity and bone integration, which may compromise long-term implant stability. Therefore, this study innovatively proposes a synergistic “composition design and process adaptation” strategy. Specifically, borosilicate bioactive glasses (BSBGs) with a high B_2_O_3_ content (36 mol%), co-doped with strontium (Sr) and magnesium (Mg), were designed and systematically compared with Sr/Mg-doped silicate bioactive glasses (SBGs). Both glasses were subsequently deposited onto Ti6Al4V substrates using atmospheric plasma spraying. The results showed that the BSBG coating exhibited an initial boron release concentration of up to 116 mg/L but exhibited excellent cytocompatibility, which is likely related to the synergistic regulation of Sr, Mg, and B ions. Moreover, the BSBG coating induced Ca-P compound mineralization within 24 h, significantly faster than the SBG coating, which required a minimum of 3 days, confirming superior biomineralization kinetics. Both coatings achieved a bonding strength of 30 MPa, meeting clinical requirements. In vivo experiments confirmed that the BSBG coating significantly promoted new bone regeneration and implant osseointegration. This work not only delivers experimental validation supporting the implementation of high-boron-content bioactive glass coatings but also provides a practical method for designing rapidly degradable and highly bioactive coatings to facilitate improved osseointegration.

## 1. Introduction

Titanium alloys exhibit outstanding mechanical properties, including high strength and low elastic modulus, along with excellent biocompatibility [1,2] and corrosion resistance [3,4]. These materials provide mechanical strength and structural stability for orthopedic implants and have therefore been widely used in clinical implantable products such as spinal interbody fusion cages and femoral stems for artificial joints [5,6,7,8]. However, titanium alloys are bioinert and exhibit limited intrinsic osteoinductive activity, which may compromise rapid osseointegration with host bone tissue. For this reason, atmospheric plasma spraying (APS) is commonly employed for titanium products to deposit hydroxyapatite (HA) coatings that mimic the structure of natural bone tissue, so as to enhance the bonding between implants and surrounding tissues [9]. In addition, additive manufacturing technologies, including 3D and 4D printing, have also emerged as promising approaches for fabricating advanced biomaterials with tailored architectures and functions [10]. Extensive efforts have been devoted to optimizing APS parameters for bioactive glass coatings, with these efforts mainly relying on trial-and-error approaches [11,12,13]. Nevertheless, the significant mismatch between the coefficient of thermal expansion (CTE) of crystalline HA and that of the titanium alloy substrate [14], together with the decomposition of HA crystals induced by the high-temperature process [15], substantially reduces both bonding strength and bioactivity, which restricts the osseointegration efficiency of bone–implant interfaces.

Bioactive glass, an amorphous material, demonstrates superior osteoinductivity and osteoconductivity [16,17,18]. Through compositional modification, bioactive glasses have been engineered to degrade in the biological environment, releasing functional ions for biomineralization and thereby stimulating osteoblast differentiation and angiogenesis to mediate bone tissue regeneration. Strontium (Sr) and/or magnesium (Mg)-doped borosilicate bioactive glasses have been found to present favorable ionic functional synergy and osteogenic potential: boron in the borate system provides essential nutritional support for bone metabolism [19,20], while Sr and/or Mg modulate the degradation rate to mitigate the excessive release of boron and directly stimulate bone formation while inhibiting osteoclast activity [21]. Therefore, depositing bioactive ceramic coatings containing strontium/magnesium ions on inert titanium surfaces to establish tight chemical bonds with bone tissue is expected to achieve both “inward growth” and “fixation” of bone tissue, opening up a new pathway for enhancing the osseointegration of implants.

Generally, glasses exhibit crystallization temperatures below the phase transformation temperatures of hydroxyapatite [22]. The application of bioactive glass as atmospheric plasma-sprayed coatings on titanium alloy surfaces has attracted extensive attention from researchers [23,24,25,26], as listed in Table 1. Given the network-modifying effects of doped atoms [21,27] in glasses, regulation of mechanical and thermophysical properties can be obtained by further optimizing composition and preparation processes [28,29,30], which is conducive to achieving a balance between bonding strength and osseointegration performance. In particular, our research team has recently developed an ultrasonic spray deposition method combined with low-temperature ceramic sintering technology, which enables the preparation of uniform and thin nanocrystalline hydroxyapatite coatings with strong interface bonding. This provides a solid foundation for the current study on bioactive glass coatings. However, most APS bioactive glass coatings have focused on classical silicate systems, while studies on high-boron-content borosilicate glasses co-doped with Sr and Mg remain limited, where the microstructure, mechanical properties, and in vitro and in vivo biological responses still require systematic investigation.

In this study, we designed a high-boron-content (36 mol%) borosilicate bioactive glass co-doped with Sr and Mg (BSBG) and systematically compared it with a Sr/Mg-doped silicate bioactive glass (SBG) without boron. This analysis aims to accomplish the following objectives: establish compositional–thermophysical coordinated design rules for bioactive glass to harmonize fast ion release and strong coating–substrate bonding; reveal the uniform deposition mechanism of bioactive coatings on complex curved substrates; and elucidate the osteogenic pathway of B/Sr/Mg multi-ions to mitigate the potential cytotoxic effects associated with high-concentration boron release. Using previous research as a reference [35,36], Sr- and Mg-doped borosilicate bioactive glasses (BSBGs) containing 36 mol% B_2_O_3_, as well as Sr- and Mg-doped silicate bioactive glass (SBG), were synthesized using the melt-quenching method and further fabricated as spraying feedstock powders in this study. Coatings were deposited on Ti6Al4V via atmospheric plasma spraying, followed by structural characterization and mechanical tests. In vitro mineralization tests, cytological experiments, and in vivo experiments were conducted to evaluate bioactivity and osseointegration performance. This work is expected to provide valuable experimental and theoretical references for further research and clinical applications of bioactive glass coatings on titanium alloys.

## 2. Materials and Methods

### 2.1. Preparation of Bioactive Glass Powders

BSBG’s composition design was 6Na_2_O·8K_2_O·8MgO·16CaO·6SrO·36B_2_O_3_·2P_2_O_5_·18SiO_2_ by mole, and that of SBG was 6Na_2_O·8K_2_O·8MgO·22CaO·6SrO·2P_2_O_5_·48SiO_2_. Bioactive glasses were prepared via the melt-quenching method using carbonates, phosphates, boric acid, and silicon dioxide (AR), which were accurately weighed as designed and then thoroughly homogenized. The raw mixtures for BSBG and SBG were melted in a platinum crucible for 60 min at 1200 °C and 1400 °C, respectively, followed by quenching into ice water. The quenched glasses were then ball-milled and sieved through a 40 μm mesh, with the sieved fraction collected for subsequent experiments.

### 2.2. Atmospheric Plasma Spraying

In the animal experiments, Ti6Al4V disks (10 × 10 × 2 mm and Φ25.4 × 6 mm), tensile blocks (Φ25.4 × 25.4 mm), and metal plates (LCLP 12 III, 4 holes, both left and right, Double Medical Technology (Xiamen, China)) were used as research samples. All Ti6Al4V substrates were degreased, pickled, and then sandblasted to *R*a = (3.6~4.2) μm. Subsequently, bioactive glass powders were sprayed onto the roughened Ti6Al4V samples using an atmospheric plasma spraying system (Multicoat, Oerlikon Metco, Wohlen, Switzerland). To achieve appropriate droplet temperature and flight velocity for flying droplets, the spraying parameters were optimized based on our previous experimental experience, as listed in Table 2.

### 2.3. Characterization of Materials

#### 2.3.1. Structural Characterization

Micro-morphology was detected using a scanning electron microscope (SEM, Prisma E, Thermo Scientific, Waltham, MA, USA) with an energy-dispersed spectrometer (EDS, Ultim Max, Oxford Instruments, Abingdon, UK, automatic mode) for composition analysis. In composition tests, five random detection points were picked for each coating (*n* = 5).

The thickness of coatings was measured by choosing five points from the cross-section morphology, and the porosity was analyzed using ImageJ software (version 1.46r) according to five random SEM images (*n* = 5).

The particle size distribution was analyzed via laser diffraction carried out using a laser diffraction particle size analyzer (Mastersizer 3000, Malvern, Malvern, UK).

The surface roughness of the coatings was measured via the stylus profilometry method (SJ-410, Mitutoyo Corporation, Kawasaki-shi, Japan). Five parallel pieces were tested (*n* = 5).

Coating structure was analyzed via X-ray diffraction (XRD, MiniFlex 600, Rigaku, Tokyo, Japan), with Cu Kα radiation (*λ* = 1.54 Å) at 40 kV and 30 mA. The test angle(2*θ*) ranged from 20° to 60° with a step size of 0.02° and a scanning speed of 5°/min.

The infrared spectral characteristics of the coatings were recorded using a Fourier transform infrared spectrometer (FTIR, Nicolet iS50, Thermo Scientific, USA) equipped with an ATR diamond crystal, in the wavenumber range of 4000~500 cm^−1^.

#### 2.3.2. Coefficient of Thermal Expansion Test

For the CTE test, BSBG and SBG powders were first sintered at 560 °C and 620 °C for 60 min, respectively, and then processed into Φ10 × 15 mm cylinders. The linear CTE of the cylinders was measured axially using a CTE tester (PCY, Xiangtan Instrument, Xiangtan, China), while Ti6Al4V cylinders with identical dimensions were simultaneously tested for comparison.

#### 2.3.3. Bonding Strength

A tensile test was used for bonding strength and failure mode analysis. A Ti6Al4V tensile block was bonded to a coated surface using an FM 1000 polyamide–epoxy adhesive film and cured at 196 °C for 3 h. A tensile test of six parallel samples (*n* = 6) was performed on a universal material testing machine (LD26.205, Lishi (Shanghai) Instruments, Shanghai, China) at a constant crosshead speed of 2.5 mm/min until coating failure occurred.

#### 2.3.4. In Vitro Self-Mineralization Test

Ti6Al4V disks of Φ25.4 × 6 mm with glass coatings were immersed in a Tris-HCl buffer solution (pH = 7.4) at 37 °C for 1, 2, 3, 7, and 14 days to evaluate in vitro self-mineralization ability. The formation rate of the bone-like apatite layer was determined to assess the bioactivity of the coatings.

#### 2.3.5. Ion Release Test

The degradation behavior of the bioactive glass coatings was evaluated by immersing samples in the Tris-HCl solution at an extraction ratio of 1 cm^2^/mL at 37 °C for 28 days. The solution was refreshed every two days during the experiment period. The concentration of the released ions was measured using inductively coupled plasma mass spectrometry (ICP-MS, Agilent 7800, Agilent, Santa Clara, CA, USA). Three parallel samples were established for each group (*n* = 3).

### 2.4. Cytological Experiments

The control groups were bare Ti6Al4V pieces (Φ25.4 × 6 mm), and the experimental groups were identical pieces with sprayed BSBG and SBG coatings. Three parallel samples were created for each group (*n* = 3) in these cytological tests. Rat bone marrow mesenchymal stem cells (rBMSCs, purchased from Baio Bowei Biotechnology Co., LTD, Beijing, China) were used for all cytological evaluations.

#### 2.4.1. Cell Culture

The rBMSCs were cultured in cell culture flasks using DMEM-F12 medium (containing 10% fetal bovine serum, 100 U/mL penicillin, and 100 μg/mL streptomycin) at 37 °C under 5% CO_2_ conditions. When confluence reached 75~90%, cells were passaged using trypsin–EDTA digestion. All experiments utilized rBMSCs from the 3rd to 7th passages.

#### 2.4.2. Biocompatibility Assay

The cytotoxicity of the samples was tested via live/dead cell fluorescent staining. Sample extracts were prepared at an extraction ratio of 3 cm^2^/mL for 24 h. The rBMSCs were seeded in 6-well plates with 2 mL of cell suspension added to each well, and the plates were cultured at 37 °C in a 5% CO_2_ cell incubator for 24 h. The original culture medium was then replaced with the prepared extracts, and the cells were cultured for 1, 4, and 7 days. At each time point, the plates were taken out successively, and the extracts were discarded. Cells were rinsed with PBS, stained with live/dead reagent, and incubated in the dark for 20 min. Cell viability was observed and photographed under a fluorescence microscope.

The rBMSCs were seeded into 24-well plates at a density of 5.0 × 10^4^ cells per well and cultured at 37 °C in a 5% CO_2_ atmosphere for 1, 4, and 7 days. At each time point, cells were transferred to new 96-well plates, cultured for another 24 h, and rinsed three times with phosphate-buffered saline (PBS). Cell proliferation was determined using the CCK-8 kit. Briefly, 180 μL of culture medium and 20 μL of CCK-8 solution were added to each well, followed by incubation for 1 h. The absorbance value at 450 nm was then measured using a microplate reader.

#### 2.4.3. Cell Differentiation Assay

For the cell differentiation samples at the predetermined time points (7 and 14 days), TRIzol^®^ reagent was added to lyse the cells on ice for 10 min. Total RNA was extracted, and a cDNA synthesis kit was subsequently used to synthesize cDNA templates for real-time quantitative reverse transcription polymerase chain reaction (RT-qPCR). The expression levels of osteogenic differentiation-related genes were detected via RT-qPCR, including Runt-related transcription factor 2 (Runx2), osteoblast-specific transcription factor (Osterix), osteopontin (OPN), alkaline phosphatase (ALP), osteocalcin (OCN), and type I collagen (Col I). The corresponding primer sequences are listed in Table 3.

Regarding the statistical analysis, the relative quantitative method (2^−ΔCT^ method) was used for the relative quantitative analysis of the amplification results to calculate the relative expression levels of each gene. One-way analysis of variance (ANOVA) combined with multiple comparisons was performed using statistical software SPSS (version 27.0.), and *p* < 0.05 indicated a statistically significant difference.

### 2.5. Animal Experiments and Surgical Procedure

A bone defect model was established in the tibia of Labrador Retrievers via osteotomy. The Ti6Al4V locking plate systems coated with BSBG coating were selected for fixation to simulate clinical application. Bare identical Ti6Al4V systems served as the control group. Six adult male Labrador Retrievers (~2.5 years old, 36.8 kg~41.6 kg) were randomly divided into 2 groups (*n* = 3 per group, randomized according to body weight and age). The dogs underwent overnight fasting or fasted for at least 6 h prior to animal anesthesia; all surgical procedures were performed under general anesthesia and strictly adhered to aseptic principles. Before anesthesia, intramuscular injection of Zoletil (3.5 mg/kg) and xylazine (0.2–1.0 mg/kg) was used to induce sedation and anesthesia in the animals, after which the surgical area was prepared and a venous channel was established. Before intubation, propofol was slowly administered intravenously to assist in anesthesia induction. The dosage of propofol should not exceed 6 mg/kg. After successful intubation of the animal, it was quickly transferred to the operating table, connected to a respiratory anesthesia machine, and maintained with 0.5% to 5% isoflurane inhalation anesthesia. The animal was placed in a prone position on the operating table and connected to a monitor. Depending on its needs, a ventilator can be used during the operation to change the breathing pattern of the animal.

Preoperative intramuscular injection of ceftriaxone sodium was administered at a dosage of 40–50 mg/kg to prevent infection, while preoperative intramuscular injection of sufentanil and meloxicam was administered at doses of 0.2–0.5 μg/kg and 0.1–0.2 mg/kg, respectively, for analgesia. Before surgery, the surgical area was disinfected and prepared, and sterile treatment towels were used.

During the surgery, physiological saline was administered intravenously to the animal, and the rate of intravenous fluid replacement was adjusted and recorded according to the animal’s condition. During the surgery, heart rate, respiratory rate, body temperature, end-expiratory CO_2_ concentration, and blood oxygen saturation were monitored and recorded at least once every 15 min. Animals were held by hand, and anesthesia was induced by an intravenous injection of propofol (5 mg/kg). Postoperatively, all animals received individual feeding with regular high-protein feed and daily intramuscular injections of cefotaxime sodium for 5 days until no abnormality was found during free movement.

After the experiment period of 8 weeks, tibial specimens were harvested, fixed in 10% neutral buffered formalin, decalcified, embedded in paraffin, and longitudinally sectioned at a 5 μm thickness. Sections were stained with hematoxylin–eosin (HE) and Masson’s trichrome. The bone area (B.Ar.) was defined as the total new tissue area within the defect region, and bone volume fraction (BV/TV) was calculated as the ratio of mineralized bone area to total callus area. Three consecutive sections per sample were measured and averaged (*n* = 3), and the intergroup differences were analyzed using one-way ANOVA.

### 2.6. Statistical Analysis

Data are presented as mean ± standard deviation (SD). Statistical analyses were performed using SPSS (version 27.0.) Differences were considered statistically significant when * *p* < 0.05.

## 3. Results and Discussion

### 3.1. Characterization of Glass Powders

**Figure 1:**
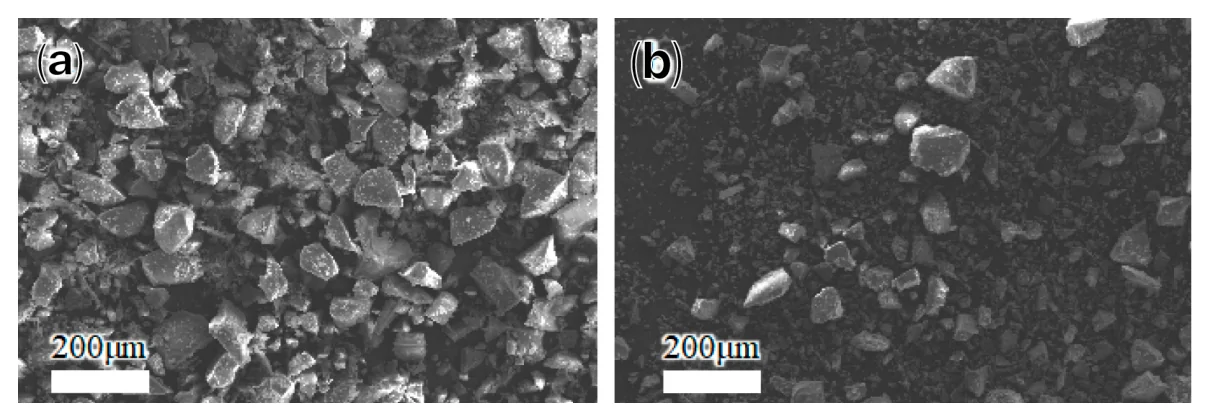
SEM morphology of (**a**) BSBG and (**b**) SBG powders.

Particle size distribution data of BSBG and SBG feedstock powders are summarized in Table 4. Overall, smaller particle dimensions were obtained for SBG compared with BSBG. Unexpectedly, the *D*_v_(100) of sieved powder was considerably larger than the nominal aperture size of the 40 μm mesh sieve employed for powder classification. Microscopic morphologies of the two powder batches are presented in Figure 1, where irregular particle shapes were identified for both formulations. Therefore, particles passing through the sieve aperture may still possess relatively large linear dimensions along certain axial directions. In addition, the micrographs revealed a narrow size distribution for BSBG particles, while a mixture of fine and coarse particles was observed in the SBG powder system [24].

### 3.2. Microstructure of Glass Coatings

**Figure 2:**
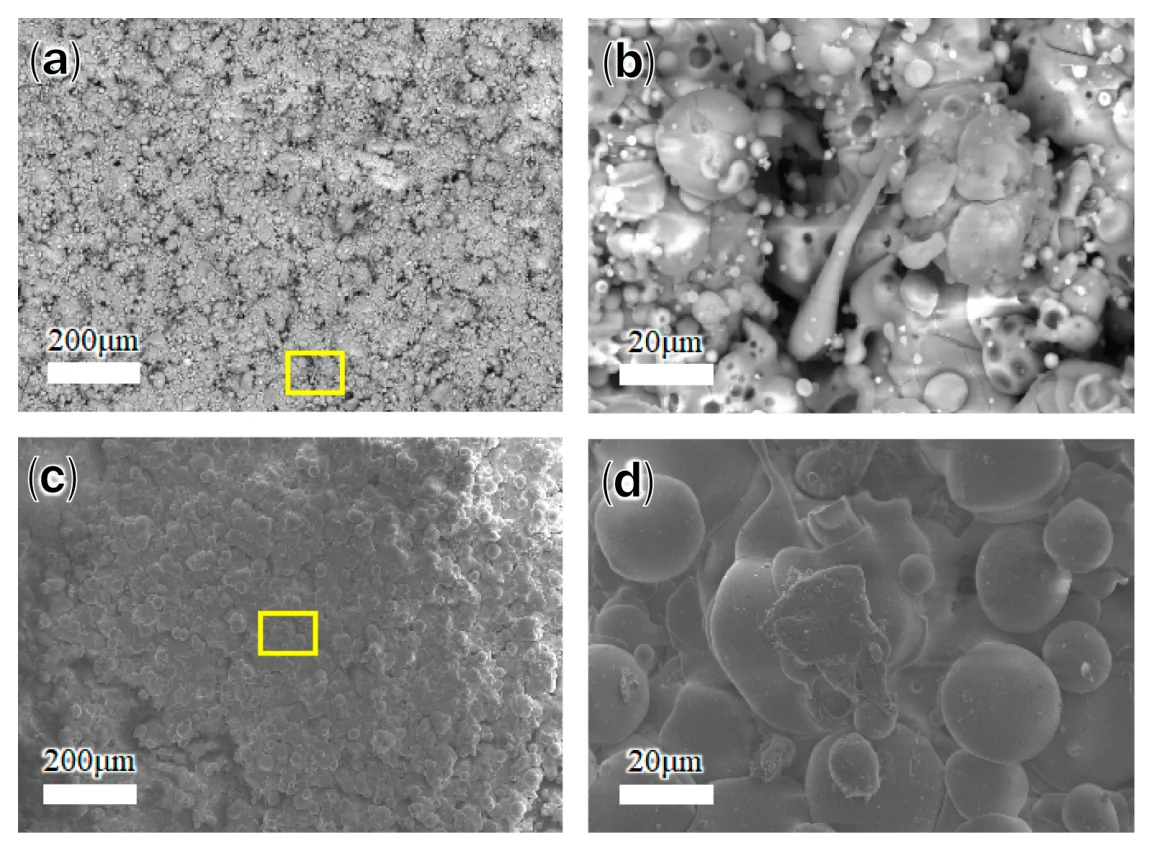
SEM morphology of (**a**,**b**) BSBG and (**c**,**d**) SBG coatings on Ti6Al4V. The yellow rectangular regions in (**a**) and (**c**) indicate the areas magnified to obtain the high-magnification micrographs shown in (**b**) and (**d**), respectively.

Figure 2 shows the micro-morphology of the bioactive glass coatings sprayed on the titanium substrate. The surfaces of both coatings exhibited a typical rough morphology formed by the spreading and stacking of molten droplets during plasma spraying. As seen in the magnified details of the yellow rectangular boxes in Figure 2b,d, the substrate surfaces were completely covered by the glasses. A few shallow microcracks were found on the BSBG coating, which is typical for APS glass coatings, and the rough microstructure increased the specific surface area of both coatings [26,31]. The fully melted droplets impacted the substrate, solidified, and accumulated to form a layered structure, producing a flattened disk-like morphology. No traces of droplets rebounding or splashing were observed, demonstrating a fine deposition process [13].

**Figure 3:**
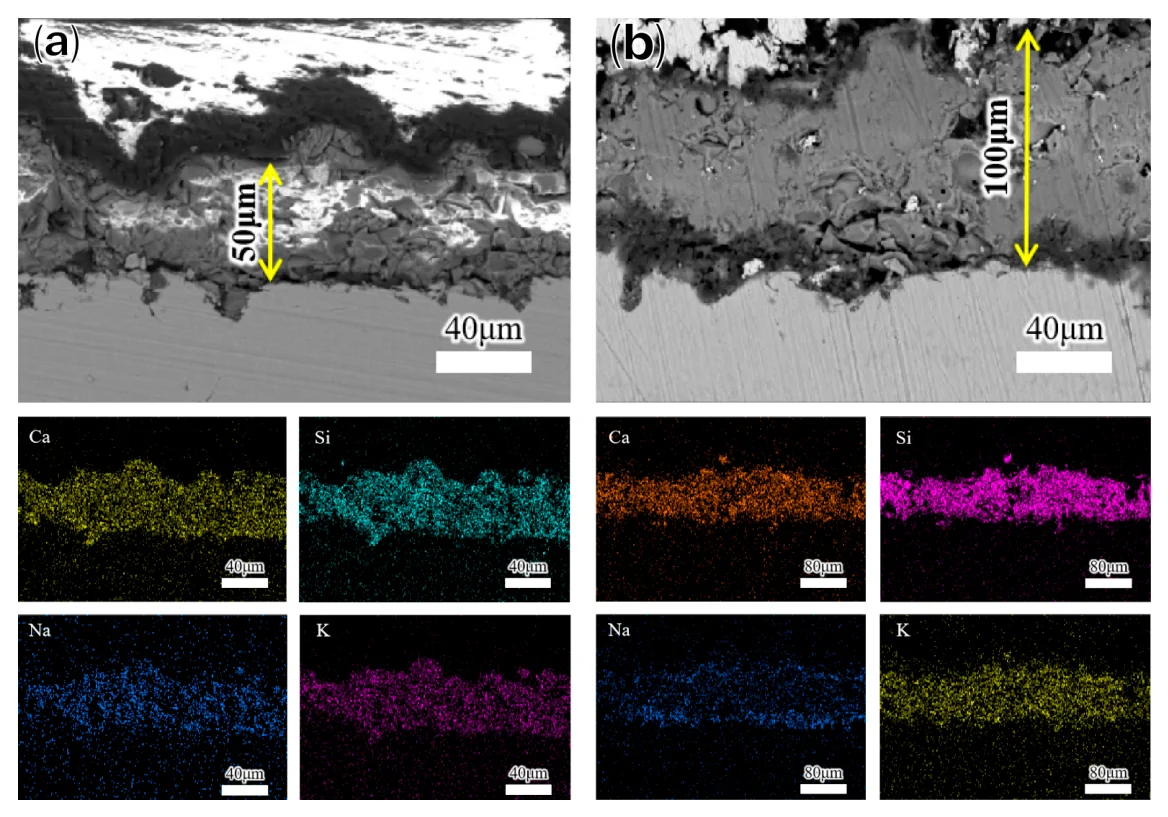
Cross-sectional morphology and element distribution of (**a**) BSBG and (**b**) SBG coatings.

The cross-sectional morphology of both coatings is shown in Figure 3, and the surface roughness (*R*a), thickness and porosity calculations can be found in Table 5. Both coatings shared similar surface roughness and porosity; no through-thickness cracks were observed in either coating. Such microstructures are typical for bioactive glasses produced via APS. Interlayer voids and microcracks observed on cross-sections originated from rapid solidification of molten splats during spraying. Such internal architectures facilitated permeation of aqueous media into coating interiors, accelerating glass matrix corrosion and subsequent apatite mineralization [31]. Several indentations were also revealed on the substrate surface, likely caused by sandblasting. The sprayed glasses filled the indentations without residual voids and thus achieved tight adhesion.

The EDS details are summarized in Table 6. Metallic elements remained largely consistent with the nominal glass formulation after atmospheric plasma spraying. A noticeable decline in potassium content was identified in both coatings, which originates from mild volatilization of low-melting alkali species under high-thermal-plasma conditions. Due to the inherent detection limitation of EDS for light elements, no boron was observed in the BSBG coating. However, the boron concentration in ICP-MS ion release tests (Section 3.5.2) indicated that boron remained incorporated in the coating and could be released into the surrounding medium. No severe selective evaporation of key osteogenic ions occurred during spraying, and the core ion delivery function of the glass system was well maintained.

A homogeneous distribution of Ca, Si, Na, and K elements is shown in both coatings (Figure 3). Generally, the differential evaporation of components at molten temperatures will lead to composition deviation. In particular, Filho [37] and Zhang [35] et al. noted that devitrification and/or precipitation may occur during quenching and spraying for Na_2_O-CaO-P_2_O_5_-SiO_2_ glasses. On the other hand, Garrido et al. [24,29,36] found that modification with doped elements can expand the amorphous phase region of the glass. In this study, both coatings sustained uniform composition, without precipitation. It is expected that the presence of B, Mg, and Sr contributed to the glasses aligning with the quenching and spraying processes, enabling compositional stability and amorphous homogeneity after deposition onto the substrate.

### 3.3. CTE Comparisons

**Figure 4:**
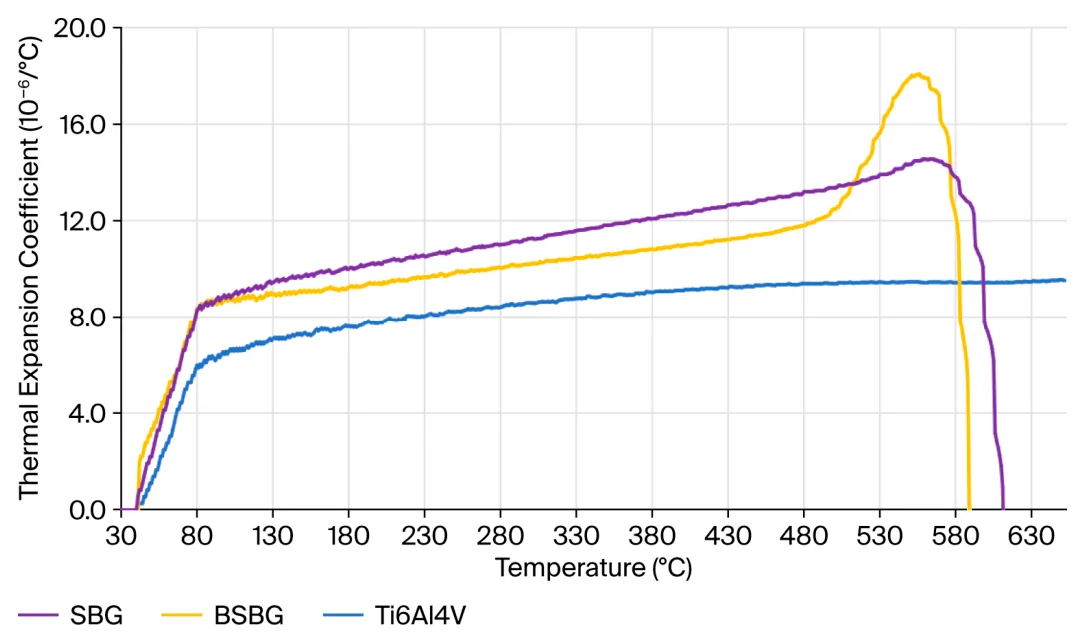
CTE–temperature curves of bioactive glasses and Ti6Al4V.

As shown in the CTE–temperature curves in Figure 4, the intensive nonlinearity of both glasses between 500 and 600 °C illustrates the glass transformation and softening regions. Moreover, the difference in thermal expansion behavior between BSBG and SBG may be associated with the high boron content in BSBG, which modifies the glass network structure by altering the boron coordination environment involving [BO_3_] and [BO_4_] units within the nonlinear range [38,39]. The average CTEs of these two glass materials (before softening) and Ti6Al4V are listed in Table 7.

Since the CTEs of both glasses prepared in this study were higher than that of Ti6Al4V, residual tensile stress was expected to develop at the glass/Ti6Al4V interface [40,41,42]. In comparison, BSBG exhibited a lower average CTE, and its high-CTE temperature range overlapped with the glass transition and softening regions, where structural relaxation and viscous flow seemed to facilitate stress relaxation. Moreover, during the cooling process, melt viscosity was gradually elevated until flow vanished. In the subsequent isothermal and co-cooling stages, glasses of lower CTE exhibited smaller shrinkage strain, matching with the substrates [18,25,40,41,42], which probably further suppressed residual thermal stress. Nevertheless, targeted research for demonstration is still required in the future.

### 3.4. Bonding Strength of the Coatings

**Figure 5:**
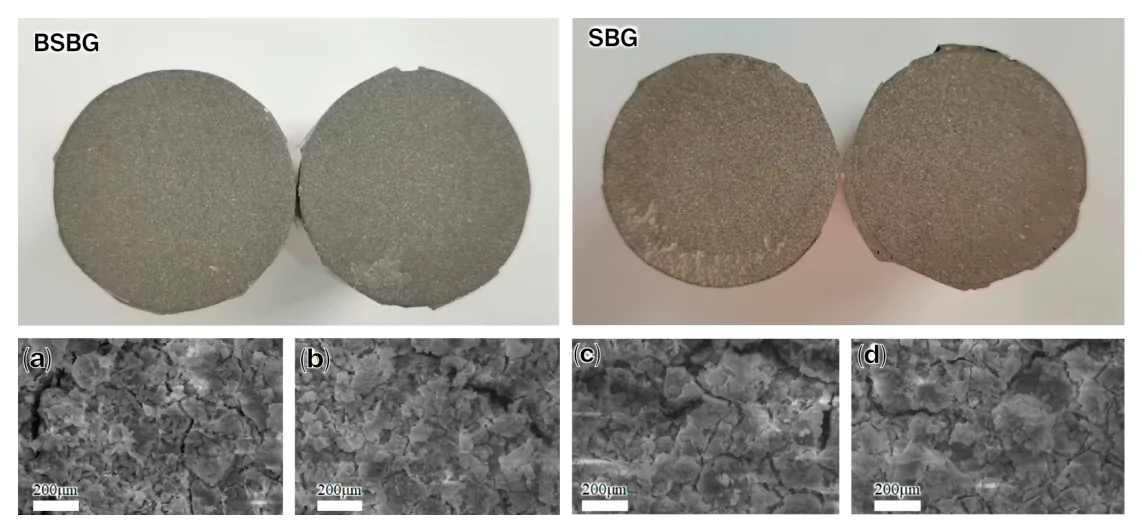
Morphology fracture interface: (**a**) BSBG coating sample and (**b**) its opposing side; (**c**) SBG coating sample and (**d**) its opposing side.

The bonding strength of the BSBG and SBG coatings reached 30.76 ± 0.97 MPa and 32.11 ± 2.26 MPa, respectively. The macroscopic photographs of the fracture surface in Figure 5 show a uniform appearance over most areas on both sides, and SEM analysis confirmed that these regions corresponded to residual glass coating. Considering the three typical failure modes of coatings—adhesive failure, cohesive failure, and mixed failure—both atmospheric plasma-sprayed coatings exhibited a cohesive failure mode rather than an adhesive kind, which revealed strong bonding strength of the samples. This was attributed to the appropriate matching between the atmospheric plasma spraying process and feedstock powder properties, resulting in the tight adhesion of the coating to the rough surface (Figure 3). On the other hand, although the CTE of SBG was slightly higher than that of BSBG, its bonding strength did not decrease, making the generally low CTEs of both glasses the dominant factor.

### 3.5. In Vitro Bioactivity Test

#### 3.5.1. Evolution of Microstructure During Self-Mineralization

**Figure 6:**
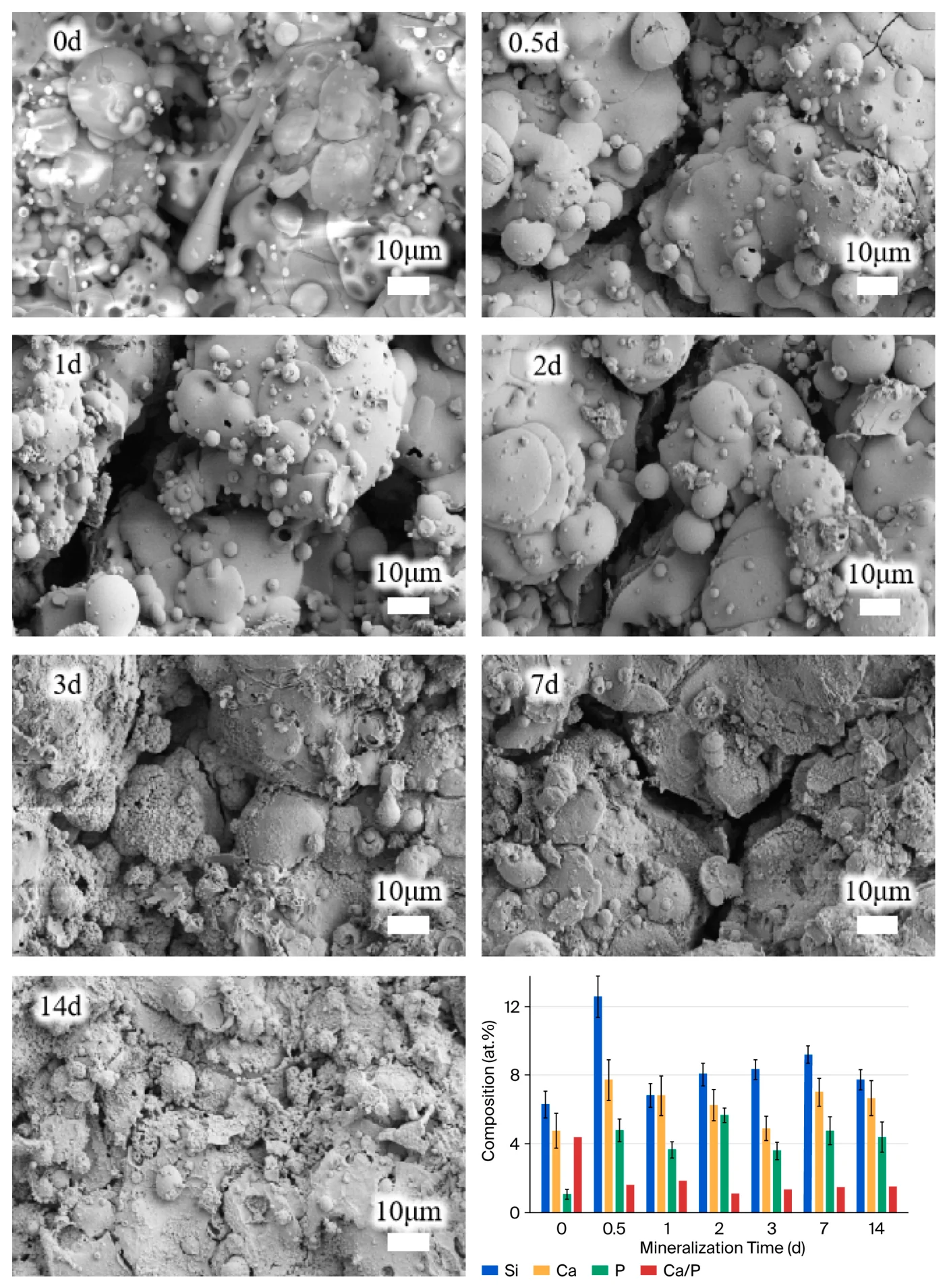
Morphology and composition evolution of BSBG coating during mineralization.

Figure 6 shows the surface morphology and composition of the BSBG coating after mineralization for different time periods. Specifically, no obvious changes were observed at day 0.5 and day 1, while spherical precipitates began to form on the surface at day 2. With the extension of immersion, the deposition on the coatings continued to grow in quantity and size, accompanied by increased surface roughness.

As the bar chart in Figure 6 shows, although the as-sprayed coating exhibited a low P content, the relative P content increased rapidly after 1 day of immersion. The Ca/P ratio dropped sharply from approximately 4 to below 2, indicating the formation of Ca-P compounds. The relative contents of Ca and P continued to increase in subsequent immersion, while the Ca/P ratio remained stable.

**Figure 7:**
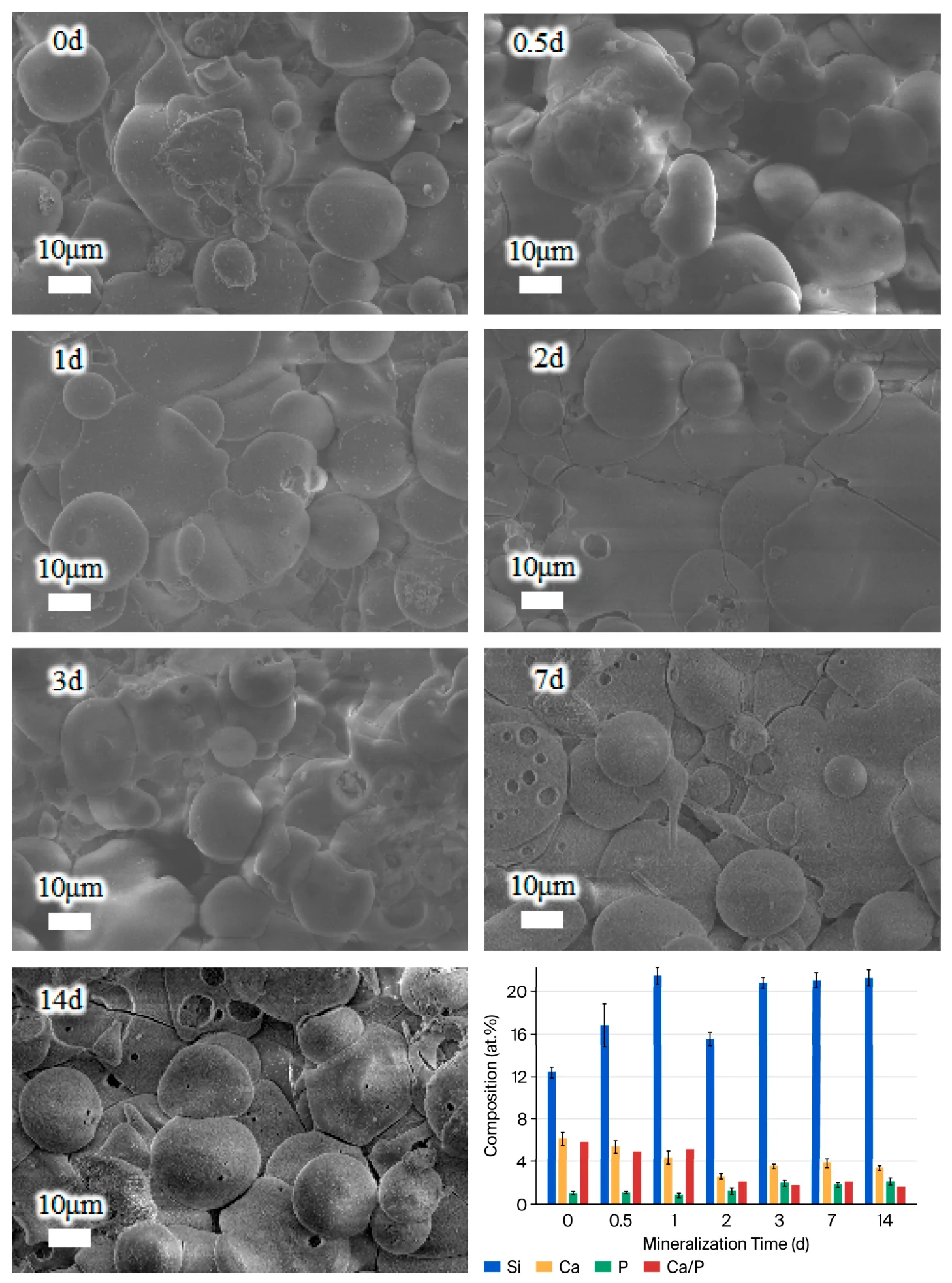
Morphology and composition evolution of SBG coating during mineralization.

For the SBG coating in Figure 7, the cooled droplets from atmospheric plasma spraying appeared to remain for 3 days, and deposits were observed at day 7. The variations in Si, Ca, and P contents of the SBG coating followed a similar trend to those of the BSBG coating but occurred at a slower rate.

**Figure 8:**
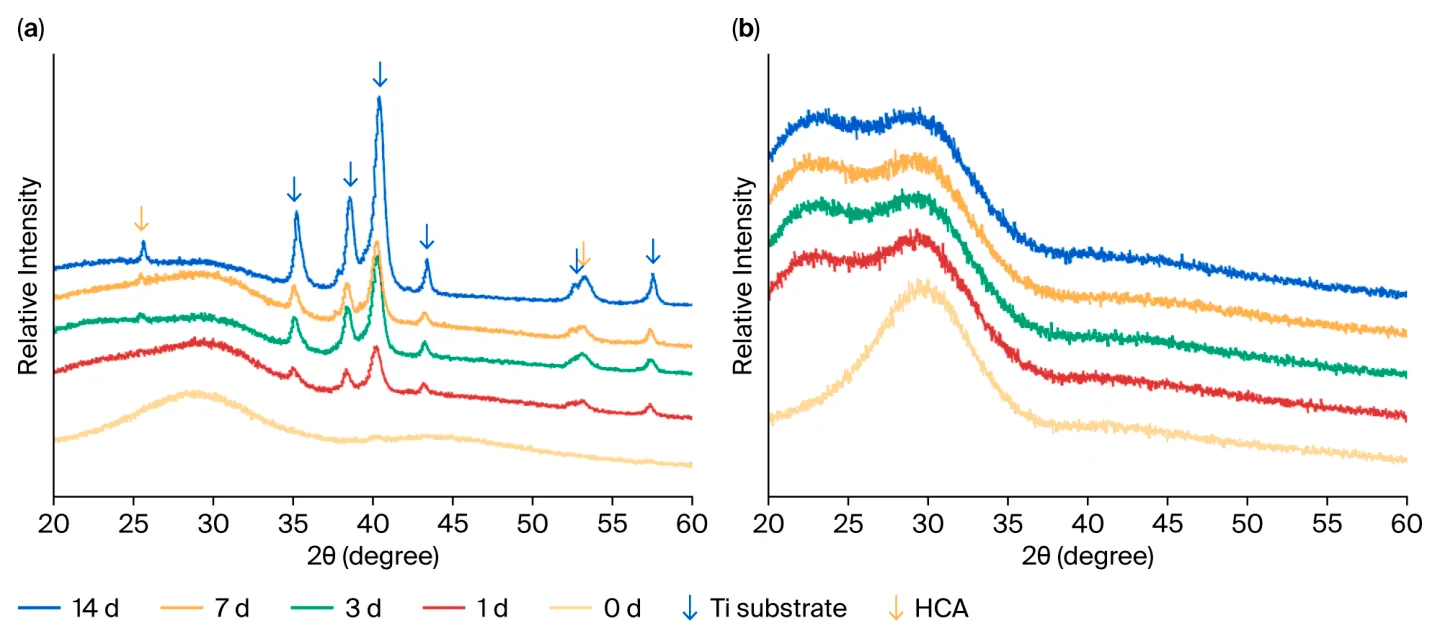
Phase evolution of the (**a**) BSBG and (**b**) SBG coatings during the mineralization test.

Figure 8 (0 d) shows the XRD results for both as-sprayed coatings. The broadened diffraction peaks near 30° confirmed an amorphous structure. Regarding the patterns of BSBG coatings shown in Figure 8a, the amorphous intensity gradually weakened with increasing immersion time, while diffraction peaks from the Ti substrate intensified, indicating degradation of the coating. Meanwhile, characteristic peaks of hydroxycarbonated apatite (HCA) at 25.6° and 53.5° indicated the growth and crystallization of the apatite layer [43]. For SBG illustrated in Figure 8b, throughout the entire week of immersion, the SBG coating surface maintained an amorphous phase, which gradually decreased, while no crystalline phase was detected.

**Figure 9:**
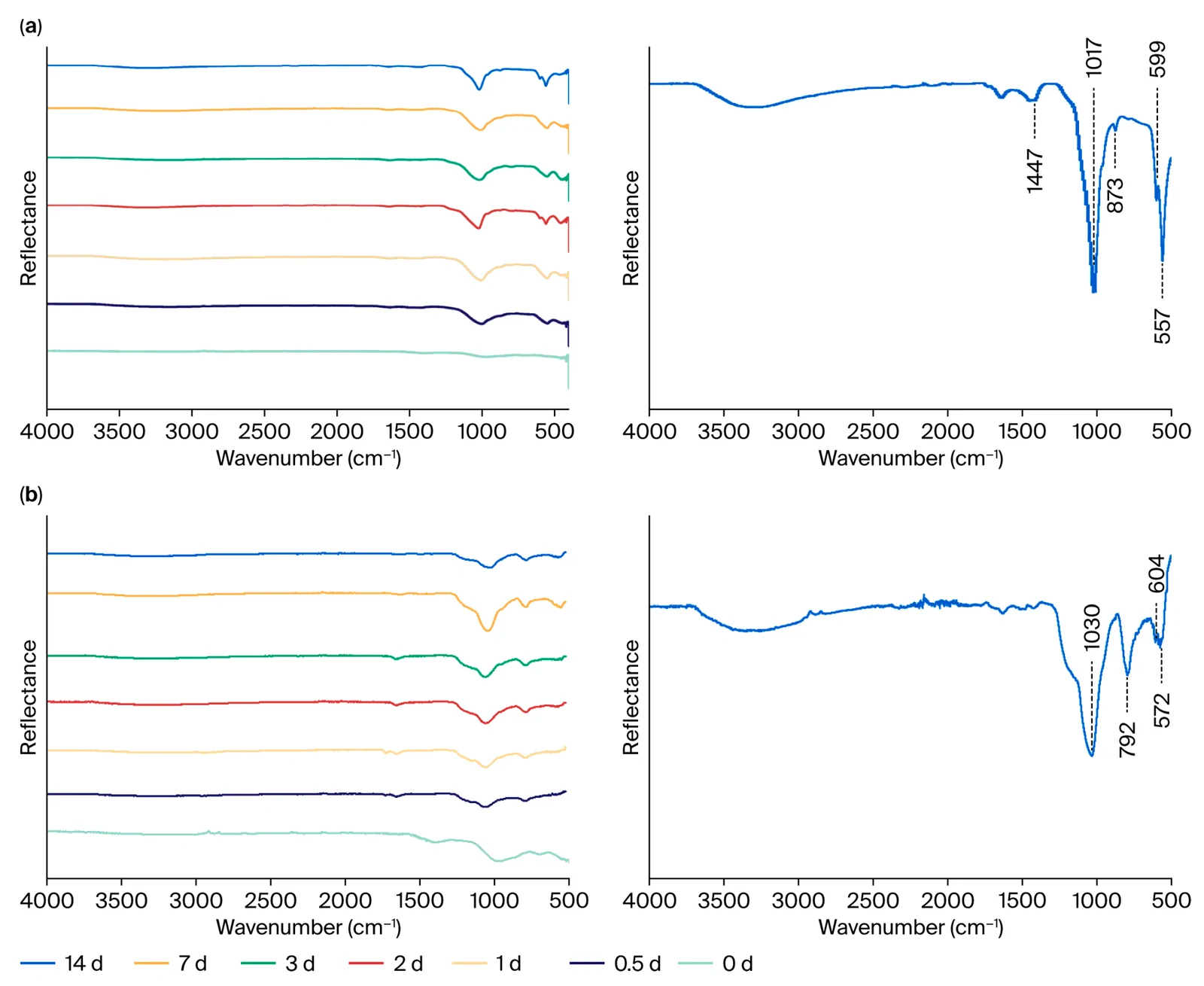
FT-IR spectra of different mineralization times for (**a**) BSBG and (**b**) SBG coatings.

The FT-IR spectra of BSBG and SBG coatings (Figure 9) captured the sequential structural evolution of glass networks during immersion, consistent with the morphological and structural characteristics described above. The broad absorption band near 1010 cm^−1^ of BSBG and SBG coatings (0 d) assigned to Si–O–Si bridging stretching of the pristine glass network gradually declined in intensity [44,45], while within the mineralization period, P–O vibration peaks (near 560 cm^−1^, 600 cm^−1^, and 1020 cm^−1^) characteristic of the PO_4_^3−^ group were detected to be gradually sharpened in both groups, which was more obvious in the single curve of 14 d. In addition, C-O vibration peaks characteristic of CO_3_^2−^ near 1447 cm^−1^ and 873 cm^−1^ were only detected in the spectra of the BSBG coating [46]. Overall, both BSBGs and SBGs induced Ca and P compounds to form apatite after immersion in Tris-HCl solution. Both materials exhibited good bioactivity, with BSBG demonstrating comparatively higher activity.

#### 3.5.2. Ion Release Rate of the Coatings

**Figure 10:**
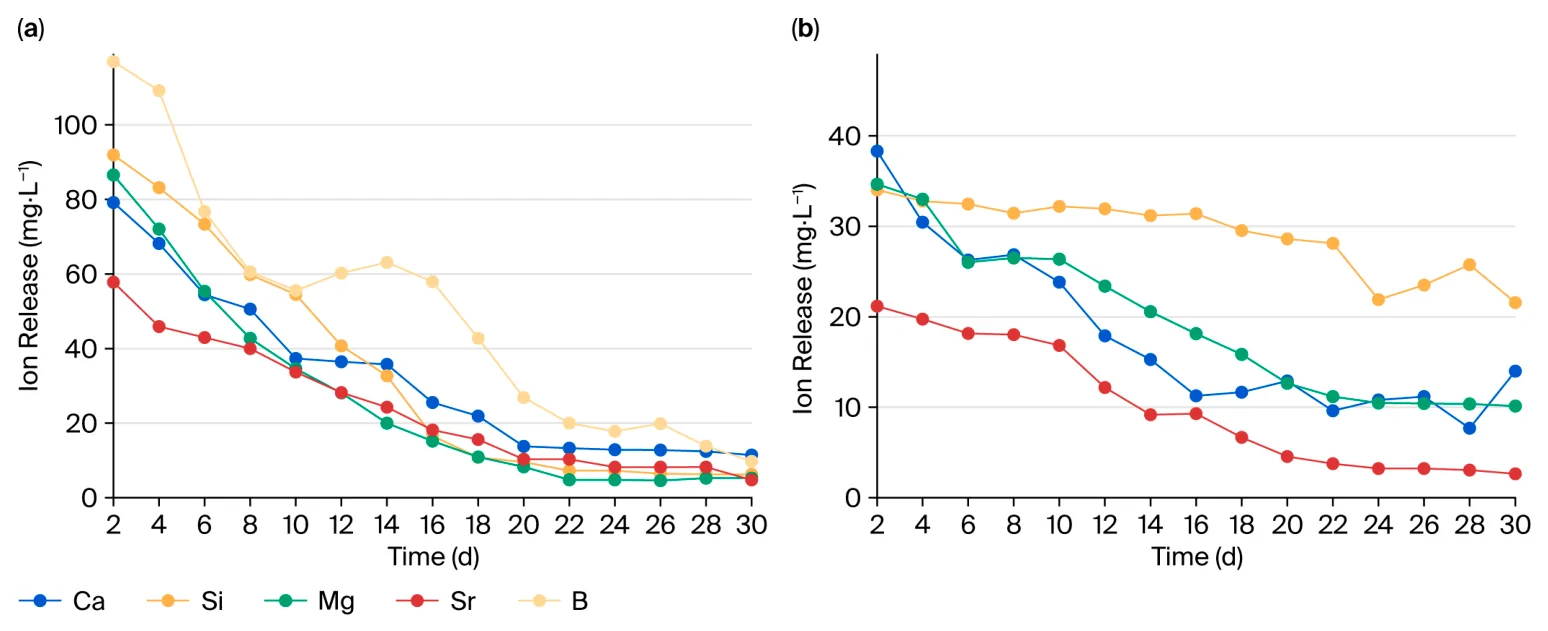
Ion release rate of (**a**) BSBG and (**b**) SBG coatings.

When immersed in a Tris-HCl solution, self-mineralization required active ions released from the samples. Ion release curves of the BSBG coatings are presented in Figure 10a. Si, Mg, Ca, and Sr exhibited similar release kinetics, characterized by an initially rapid release rate followed by a gradual decrease, which may result from the progressive consumption of soluble ions and the formation of a mineralized layer that hindered further ion diffusion. In contrast, the release level of B exhibited a mild rebound in the second week, which might require further investigations regarding its kinetic mechanisms. For the SBG coatings (Figure 10b), Ca, Sr, and Mg showed rapid release during the first week, followed by a gradual decrease with fluctuations. Si maintained a stable release for almost 30 days. Overall, the cumulative ion release amount of BSBG was almost twice that of SBG.

These release characteristics and differences originated from the compositional differences between the two glasses. The primary distinction lies in the high boron content of BSBG, while SBG was boron-free but silicon-rich. Although both are network-forming units, silicon provided stronger stabilization (Si–O: approximately 452 kJ/mol; B–O: approximately 301 kJ/mol) [19,20]. Thus, the enhanced ion release of BSBG was mainly driven by boron-mediated depolymerization of the glass network. Given the lower bond dissociation energy of B–O linkages, the cross-linking degree of the amorphous skeleton declined substantially and expedited matrix erosion in aqueous environments. Even with glass framework of rapid ion release, the coating sustained mechanical adhesion equivalent to SBG. This favorable outcome stemmed from tuned thermophysical characteristics of boron: reversible transformation between [BO_3_] and [BO_4_] units in the glass transition range enabled viscous flow relaxation, which alleviates thermal expansion mismatch with Ti6Al4V substrates [38,39]. Collectively, the compositional design dictated glass topological configurations and thermal responses, which jointly governed the degradation rate and interfacial mechanical reliability.

It has been well documented that higher concentrations of Ca^2+^ favor the deposition of the Ca-P compound. Furthermore, the release of bioactive elements, including Ca and Si, under physiological implantation environments, has been verified to accelerate the formation of mineralized bone architectures by osteoblasts [47]. Accordingly, the accelerated ionic release from BSBG coatings may contribute to their enhanced osteointegration capability.

### 3.6. In Vitro Cytological Characterization

#### 3.6.1. Live/Dead Cell Fluorescent Staining

**Figure 11:**
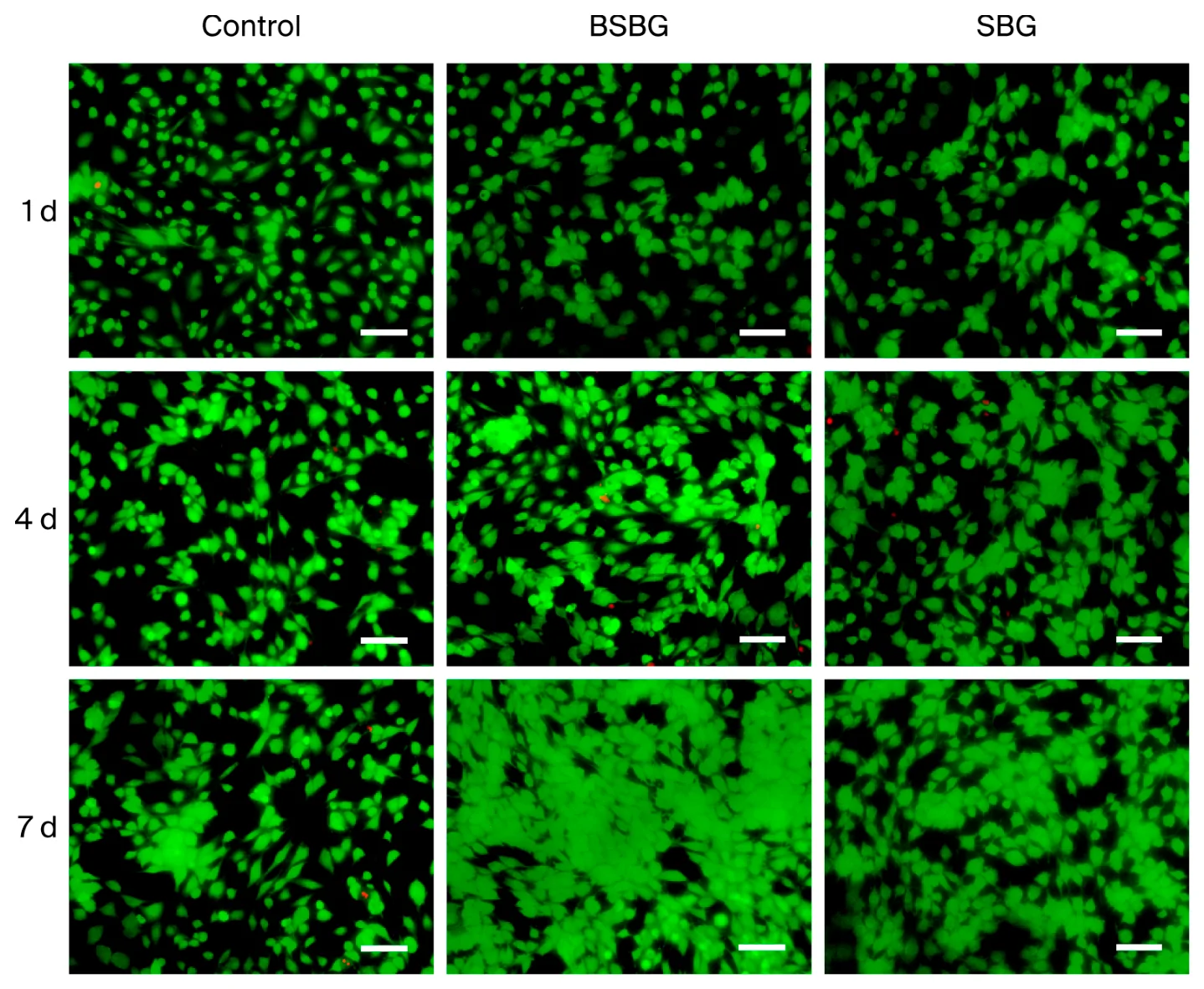
Fluorescent live/dead staining image of rBMSCs (scale bar: 100 μm, green: live, red: dead).

Fluorescent live/dead staining results are shown in Figure 11. At day 4, an increase in viable cells was observed in the experimental groups, while no significant change was noted in the control group. After 7 days of culturing, the number of viable cells in the experimental groups markedly increased, while the number of dead cells remained low across all groups at all three time points.

#### 3.6.2. Cell Proliferation

**Figure 12:**
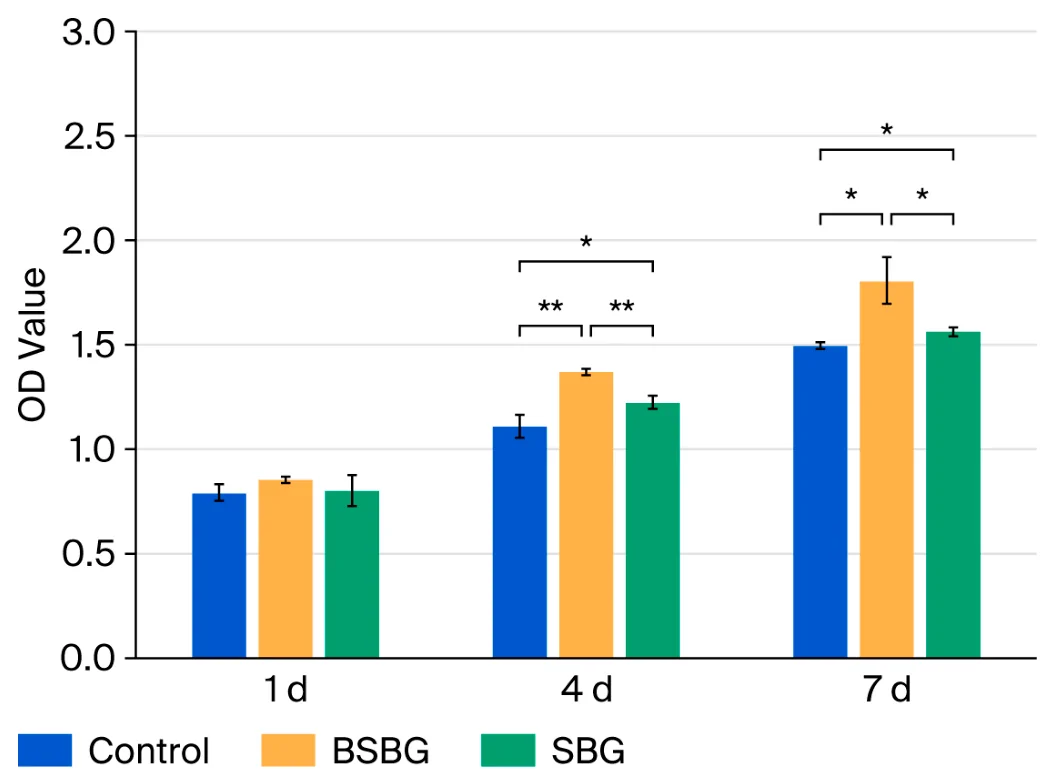
OD values of the CCK-8 assay for rBMSCs (* indicates a significant difference between the two groups of samples, with * *p* < 0.05 and ** *p* < 0.01).

From the cell proliferation graph in Figure 12, at day 1, the OD values were similar between the control and experimental groups. After 4 days of culturing, the BSBG group exhibited the highest OD value, while SBG showed a slightly higher value than the control group. This trend was sustained until day 7. No cytotoxicity was observed in the experimental groups. Compared to the Ti6Al4V substrate, the bioactive glass coatings promoted cell proliferation, with the BSBG coating exhibiting a more pronounced effect. These results confirmed that the coatings exhibited no obvious cytotoxicity and provided a favorable effect on promoting cell proliferation, which was consistent with the live/dead staining results.

#### 3.6.3. Osteoblast Differentiation

**Figure 13:**
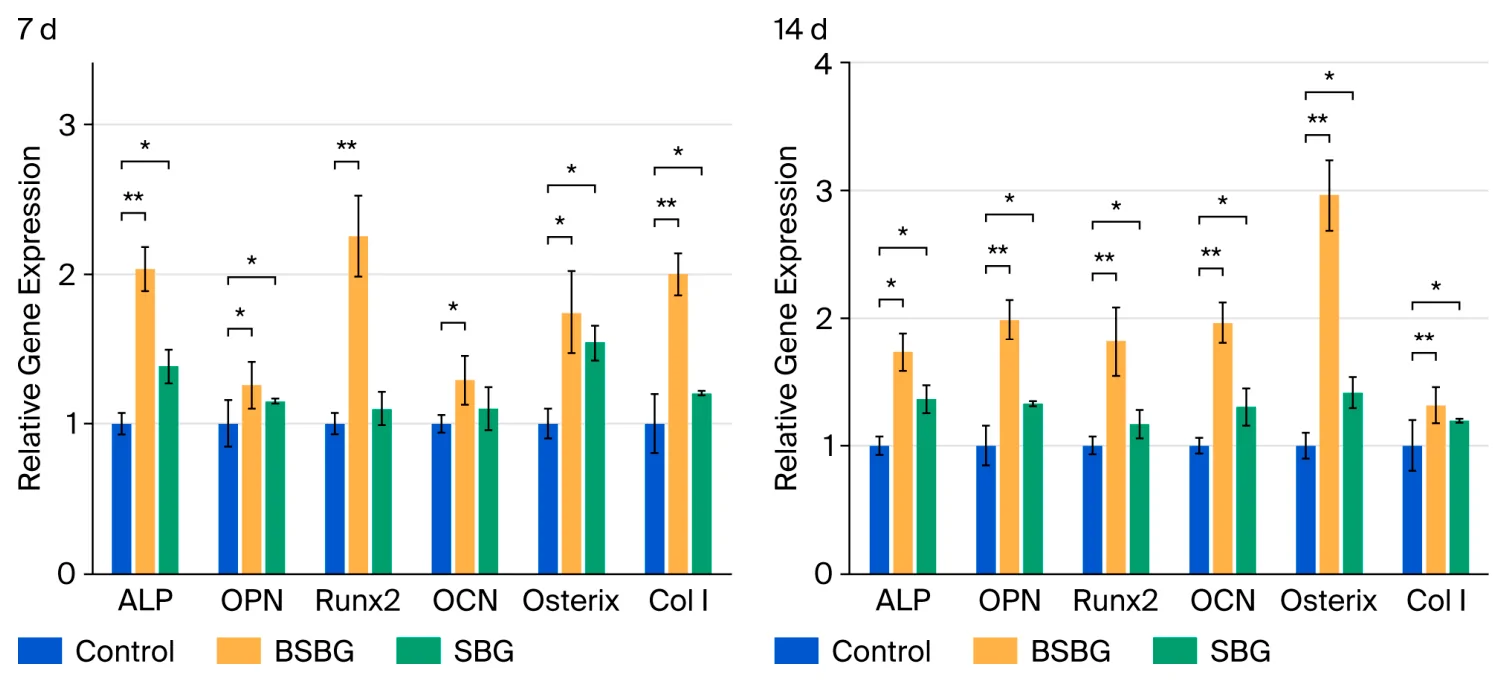
Gene expression of rBMSCs (* indicates a significant difference between the two groups of samples, with * *p* < 0.05 and ** *p* < 0.01).

Regarding the relative gene expression shown in Figure 13, after 7 days of culturing, all target genes were upregulated in the experimental groups, with significantly higher expression levels detected in the BSBG group than in the SBG group. The results at 14 days showed a similar trend to those observed at 7 days, and the BSBG coating still induced higher expression. Osteoblast differentiation is known to require specific concentrations of Ca and Si in the extracellular microenvironment. Combined with the ion release profiles, ions released from the bioactive glass coatings appeared to provide a favorable condition for cell differentiation. Simultaneously, the rapid ion release from BSBG further strengthened its stimulatory effect on osteoblast differentiation.

The compositional difference and resultant glass network variation further governed the ion release profiles, which sequentially regulate the extracellular microenvironment and downstream osteogenic gene expression of rBMSCs. Despite the mineralization effect mentioned in Section 3.5.2 above, coordination of silicon and calcium activates cascades that elevate ALP secretion and accelerate extracellular matrix mineralization, jointly promoting full osteoblast maturation [48,49]. Both magnesium and strontium tune glass compactness and degradation kinetics. Appropriate release of Mg and Sr preserves rBMSC viability, as well as regulating multiple osteogenic genes [21]. Moderate concentration of boron maintains normal rBMSC viability, elevates ALP activity and pro-osteogenic gene expression, and stimulates cell migration to assist bone repair [19,20,50,51]. In this study, the bioactive glass coatings sustained sufficient element release (Figure 9). Multi-ion release from optimized glass networks achieves regulation of rBMSC viability, early mineralization capacity, and late osteogenic gene transcription. Both coatings in this study exhibited notable bioactivity, especially the BSBG coating, which was closely associated with its higher ion release rate.

Conversely, reports on boron content showed that BMSC activity was reduced at boron concentrations above 8.5 mg/L and MC3T3-E1 viability decreased when the released boron concentration exceeded 1 mg/L [20,52]. Interestingly, the boron release of the BSBG coating reached over 100 mg/L during the early stages of degradation, yet cell experiments have confirmed the promotional effect on cell proliferation and differentiation. Similar observations have also been reported [19,20,52,53]. This may be explained by the reduction in the dynamic boron concentration exposure for cells compared to the initial sample extracts. Serum protein complexation, dynamic cell metabolism, and extracellular matrix adsorption continuously lower local boron levels surrounding cells. In addition, divalent cations stabilize cell membranes and activate osteogenic pathways, likely offsetting potential boron-related stress [19,21,50]. However, this observed cytocompatibility merely reflected the favorable biological performance of this multi-ionic degradation system rather than a shift in the cytotoxic threshold of boron for rBMSCs.

### 3.7. In Vivo Experiments

**Figure 14:**
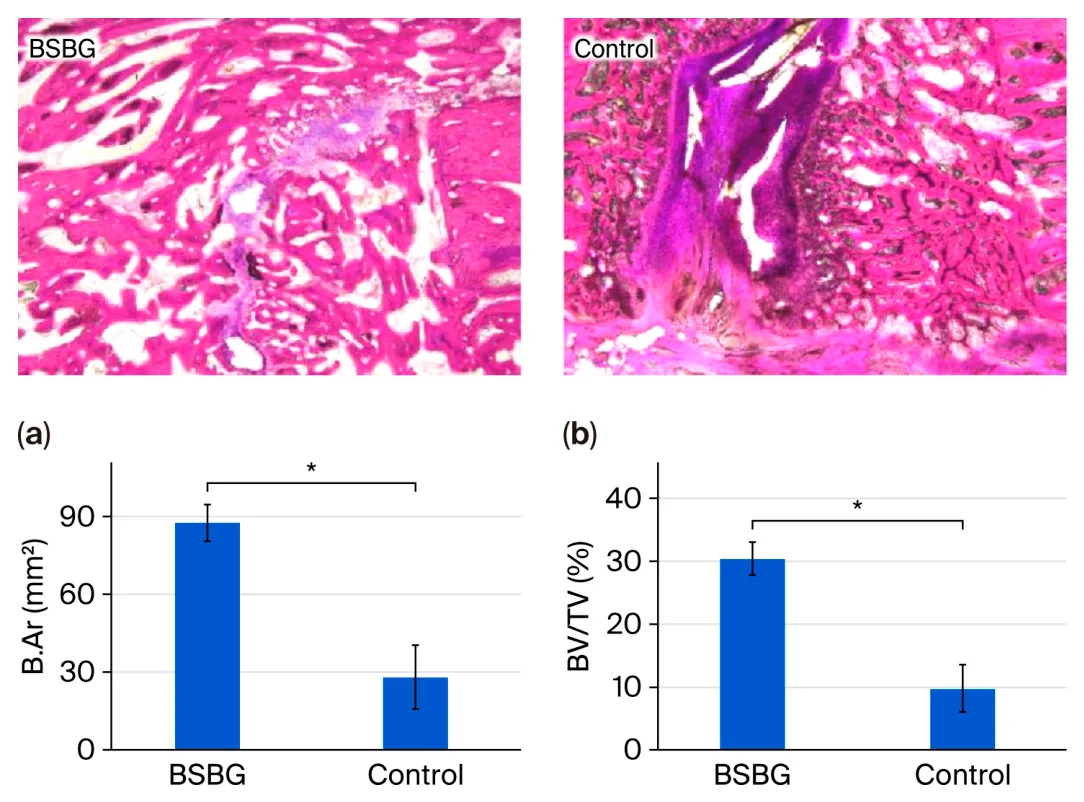
Histopathological analysis of new bone on the surface of implants: (**a**) callus area; (**b**) bone volume fraction (* indicates a significant difference between the two groups of samples, * *p* < 0.05).

Histopathological images in Figure 14 reveal that the control group exhibited limited new bone formation around the cortical bone area, whereas substantially more new bone tissue was observed in the experimental group. Quantitative histomorphometric analysis (Figure 14a) showed that the bone area (B.Ar.) in the experimental group reached 87.56 ± 6.98%, which was significantly higher than the 28.07 ± 12.32% value of the control group. The bone volume fraction (BV/TV, Figure 14b) within the callus of the experimental group was 30.38 ± 2.63%, which was higher than that in the control group (9.78 ± 3.75%). These results indicated that the BSBG coating significantly enhanced new bone formation and contributed to enhanced bone regeneration around the implant site in vivo.

## 4. Conclusions

In summary, in this study, a novel Sr/Mg-doped high-boron-content bioactive glass coating (BSBG) was successfully developed with significant improvements in material design, biological performance, and clinical potential. The main conclusions are as follows:

1. Material innovation: A borosilicate bioactive glass with 36 mol% B_2_O_3_ co-doped with Sr and Mg was designed and systematically investigated. Compared to traditional silicate glass (SBG), BSBG exhibited a lower coefficient of thermal expansion (10.5 vs. 11.3 × 10^−6^/°C), better matching Ti6Al4V and reducing residual stress. This composition breaks the conventional view that high boron content impairs stability.
2. Biological innovation: The BSBG coating induced Ca-P mineralization within 24 h, much faster than SBG (≥3 days). Despite an early boron release as high as 116 mg/L, typically considered cytotoxic, BSBG significantly promoted rBMSC proliferation and osteogenic differentiation (upregulating Runx2, OCN, and ALP). This reveals that the multi-ionic environment containing B, Sr, and Mg ions may contribute to maintaining cytocompatibility under the current experimental conditions despite the high initial boron release.
3. Application innovation: Both coatings achieved a bonding strength of ~30 MPa, indicating sufficient interfacial stability for potential biomedical applications. In vivo experiments in a canine tibia defect model confirmed that BSBG significantly enhanced new bone formation (30.38% vs. 9.78% in the control group) and promoted implant osseointegration. This work establishes a dual “mechanical interlocking and chemical bonding” strategy, offering a promising solution for high-end dental implants to achieve import substitution.

## Figures and Tables

**Table 1:** Effects of B, Mg, and Sr on the bioactivity of APS bioactive glass coatings.

Glass System	Dopants	Bonding Strength	Key Element Released	Biological Evaluation
45S5 [24,31]	-	8~10 MPa	Si, Ca, P	HCA formation within 3 days in HBSS
62W [30,31]	Mg	(32 ± 3) MPa	Mg, Si, Ca, P	HCA formation within 7 days in SBF
BG0 [32]	B, Mg, Zn	Disks	B, Mg, Ca, P, Zn	HCA formation within 7 days in SBF
LY-B3 [33]	B, Sr	Enameling, fracture toughness improved	B, Ca, Sr, P	-
BGMS10 [34]	Mg, Sr	-	Mg, Sr, Si, Ca, P	HCA formation within 7 days in SBF

**Table 2:** Atmospheric plasma spraying parameters.

Power	23.8 kW
Current	450 A
Spraying distance	100 mm
Argon flow rate (primary gas)	45 SLPM
Nitrogen flow rate (secondary gas)	5 SLPM
Torch speed	100 mm/s
Feeding rate	20 g/min

**Table 3:** Primer sequences utilized for the RT-PCR analyses.

Gene Name	Sequence (5′→3′)
Runx2-F	GAGTGGACGAGGCAAGAGTT
Runx2-R	GAGGCGGTCAGAGAACAAAC
Osterix-F	CTTGTGCCTGATACCTGCACT
Osterix-R	TCACTCTACCTGACCCGTCATC
OPN-F	CATCACCTGTGCCATACCAG
OPN-R	GTCATGGCTTTCGTTGGACT
ALP-F	GACCTCCTCGGAAGACACTC
ALP-R	TGAAGGGCTTCTTGTCTGTG
OCN-F	ATGAGGACCCTCTCTCTGCTC
OCN-R	CTAAACGGTGGTGCCATAGAT
Col I-F	CGAGCTCGGCAATGGAATCTTGGATG
Col I-R	CCGCTCGAGCGGAGGTCCACAAAGCTG
GAPDH-F	AACGGATTTGGTCGTATTGG
GAPDH-R	GTCACCGGAGTCCATCCGAT

**Table 4:** Particle size of glass powders (μm).

Powders	*D*_v_(10)	*D*_v_(50)	*D*_v_(90)	*D*_v_(100)
BSBG	5.96 ± 0.06	26.8 ± 0.10	42.4 ± 1.86	105.2 ± 2.71
SBG	2.83 ± 0.02	12.4 ± 0.21	35.8 ± 1.57	113.7 ± 2.46

**Table 5:** Thickness and porosity of both coatings.

Coating	*R*a (μm)	Thickness (μm)	Porosity (%)
BSBG	4.3 ± 0.6	51 ± 8	27.3 ± 2.1
SBG	4.8 ± 0.5	105 ± 19	25.8 ± 2.9

**Table 6:** Surface composition of bioactive glass coatings (at%).

Coatings		Na	Mg	Si	K	Ca	Sr	P	O
BSBG	Nominal composition	3.43	2.29	5.14	4.57	4.57	1.71	1.14	56.57
	Coating composition	3.39 ± 0.35	2.23 ± 0.50	6.29 ± 0.78	3.03 ± 0.42	4.54 ± 1.01	1.79 ± 0.48	1.08 ± 0.29	Residual
SBG	Nominal composition	4.41	2.94	17.65	5.88	8.09	2.21	1.47	57.35
	Coating composition	4.11 ± 0.58	2.62 ± 0.29	12.39 ± 0.49	2.51 ± 0.30	6.18 ± 0.59	1.70 ± 0.24	1.05 ± 0.16	Residual

**Table 7:** Average CTEs of glasses and Ti6Al4V/10^−6^·°C^−1^.

Materials	Ti6Al4V	BSBG	SBG
Average CTE	8.3	10.5	11.3

## Data Availability

The original contributions presented in this study are included in the article. Further inquiries can be directed to the corresponding authors.
